# A Reinforcement Learning approach to study climbing plant behaviour

**DOI:** 10.1038/s41598-024-62147-3

**Published:** 2024-08-06

**Authors:** Lucia Nasti, Giacomo Vecchiato, Patrick Heuret, Nicholas P. Rowe, Michele Palladino, Pierangelo Marcati

**Affiliations:** 1 Gran Sasso Science Institute, L’Aquila, Italy; 2 AMAP, Univ Montpellier, CIRAD, CNRS, INRAe, IRD, Montpellier, France; 3 DISIM, Department of Information Engineering, Computer Science and Mathematics, University of L’Aquila, Via Vetoio, 67100 L’Aquila, Italy

**Keywords:** Bioinformatics, Biological models, Software, Computational biology and bioinformatics, Systems biology, Mathematics and computing, Plant sciences, Plant evolution, Plant genetics, Plant physiology, Plant stress responses

## Abstract

A plant’s structure is the result of constant adaptation and evolution to the surrounding environment. From this perspective, our goal is to investigate the mass and radius distribution of a particular plant organ, namely the *searcher shoot*, by providing a Reinforcement Learning (RL) environment, that we call Searcher-Shoot, which considers the mechanics due to the mass of the shoot and leaves. We uphold the hypothesis that plants maximize their length, avoiding a maximal stress threshold. To do this, we explore whether the mass distribution along the stem is efficient, formulating a Markov Decision Process. By exploiting this strategy, we are able to mimic and thus study the plant’s behavior, finding that shoots decrease their diameters smoothly, resulting in an efficient distribution of the mass. The strong accordance between our results and the experimental data allows us to remark on the strength of our approach in the analysis of biological systems traits.

## Introduction

Plants are living organisms coordinating a complex network of internal, e.g., nutrient concentration, and external signals, e.g., light and soil resources. As a result, plant growth is a delicate balance among different factors involving environmental and physiological conditions^1^.

Despite their sessile life, plants can move and react to external stimuli to search for nutrients, and avoid obstacles and dangerous conditions^2^. In contrast to the animal kingdom, plants do not perform these movements only through “active” reversible actions, but also by expanding their organs^3^. Plants actually live in a complex environment with a limited amount of resources. These resources are shared between plants of the same species as well as plants of different species. In this context, the efficient use of such resources can be crucial for plant subsistence. For instance, in its growth process, a plant produces a limited amount of biomass. It can use such mass to elongate one of its stems (primary growth process) or to produce rigidity and stability of its organs (secondary growth). Efficient employment of biomass means finding the most convenient threshold between primary and secondary growth, considering that the longer the stem, the better the exploration, but the thicker, the more resistant it is to mechanical stress.

The concept of efficiency may be the key to understanding how plants interact with the environment and develop their organs. This gives a different perspective to plant modelling. Indeed, this point of view enriches a model by adding to the mathematical description of the biological system a possible interpretation. The possibility of mathematically studying the mechanics of a structure and understanding the extent of its physical limits has fascinated scientists since the time of Galileo (see in particular the work “Two new sciences”). Specifically on plants, there are studies on critical lengths (see for instance^4–6^), on the distribution of roots and branches^7^, or on how a root penetrates the soil^8^. The optimisation paradigm can be applied effectively in biological contexts^9,10^ and is what has stimulated this work.

**Figure 1:**
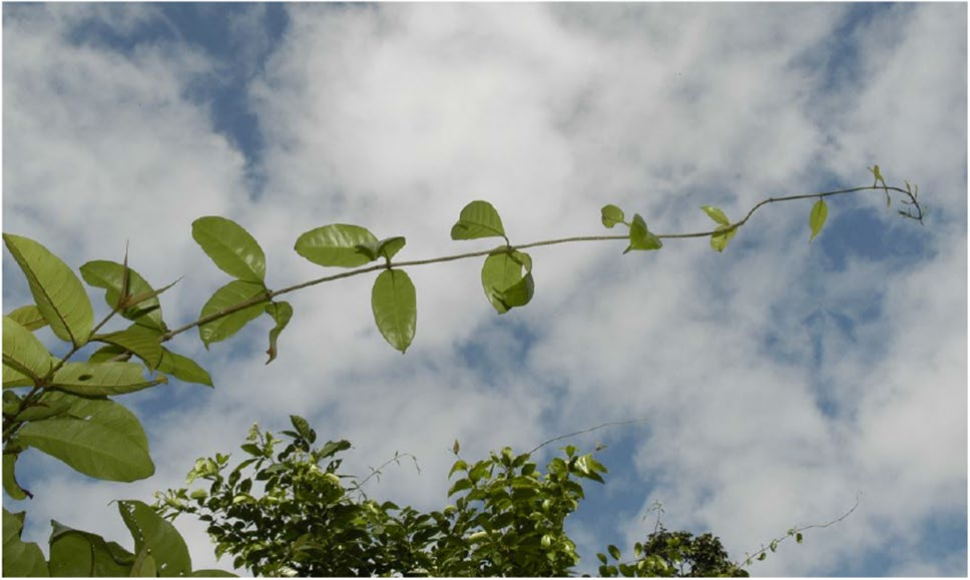
Searcher of Condylocarpon guianense in humid tropical forest canopy of French Guiana. Like many searchers the stem bears numerous expanded leaves and shows evidence of “adjustments” in stem direction along its length. In the lower part of the picture, additional searchers of the same species show the variety of searcher stem developments and orientations at different stages of development.

In this work, we focus on a particular climbing plant species, known as the *Condylocarpon guianense* Desf. (see Fig. 1).

This plant species is a liana widely found in the flora of French Guiana, which twines around the branches and the trunks of neighboring plants in order to reach the canopy. Several studies on its structure, see^11–14^ for instance, have revealed that in different growth stages, it changes the thickness and the nature of the layers that form its stem and consequently it changes its flexural rigidity. More specifically, the plant is more rigid during the self-supporting state, while it displays a less dense material and a thicker compliant cortex when attached to a support. Such a wide capability of *C. guianense* to adapt to the surrounding environment suggests that it is following a paradigm of efficiency, making it a suited subject for our study. In particular, we want to support the hypothesis that the self-supporting organs of *C. guianense*, called searcher shoots, maximize their length avoiding a maximal stress threshold. This idea is motivated by the fact that the searcher shoots are organs specialized in finding a support to attach themselves. Hence, the longer they are, the better they explore the surrounding environment and cross gaps. However, at the same time, they have to sustain their weight^15^.

To investigate this specific behavior and prove our hypothesis, we combine mechanical modelization and Reinforcement Learning (RL). Specifically, to prove that climbing plants optimize the mass distribution in their self-supporting stems, we developed a RL environment, which we called Searcher-Shoot, to study the radius along the climbing plant shoot. At the base of this environment, we considered two planar mechanical models: (MeLe) and (Me), which give us information about the development of the curvature and the mass along the shoot, considering the leaves or not, respectively.

The application of Artificial Intelligence (AI) to many biological problems is increasing rapidly in the analysis of plant morphology, growth, and development, or the understanding of their changing environment in conjunction with agriculture^16^. In particular, Machine Learning (ML) is playing a conspicuous role in developing predictive models in complex plant biological systems^17^ whenever possible the integration and the analysis of multidimensional omics data^18^. Moreover, as described in Ref.^19^, with inadequate data, AI/ML applications perform poorly. When Supervised and Unsupervised methods are not able to generate a direct linear or non-linear mapping among the raw data, RL stands up for being a valid alternative method^20,21^ and, as underlined in Ref.^22^, it is emerging as a robust and reliable tool to face out real-world problems concerning biological systems. Example of this application are available in synthetic biology^23^, metabolic engineering^24^, chemical reaction network^25^, and plant biology^26^.

Our study delved into the complex and dynamic interactions that occur within plant growth behavior. Traditional numerical methods rely on deterministic models that may not accurately represent the nonlinear and dynamic nature of plant behavior. In contrast, we utilized RL which is better suited to capture the nuances of plant responses to various stimuli as it adapts to complex, changing environments.

RL’s trial-and-error learning approach allows our model to explore and adapt to the dynamic behaviors of plants, enabling us to uncover optimal strategies that may not be evident in static, pre-defined models. Additionally, RL operates in a data-driven manner, which is particularly advantageous when studying plant dynamics. This allows us to extract patterns and behaviors that may be difficult to capture using theoretical, equation-based approaches.

To test our RL environment, we applied it to five samples of *C. guianense*, using experimental data provided by Refs.^15,27^. We found that the optimal policy was able to reproduce the decreasing behavior that characterizes the radius of the sample in consideration. This outcome suggests that, at least for *C. guianense*, the mass distribution along the searcher shoot is distributed optimally to maximize the length while avoiding the curvature reaching a certain breakdown threshold.

## Results

We develop the Searcher-Shoot environment in Python. Precisely, we employ the OpenAIGym^28^ and Stable-Baselines3 (SB3)^29^ libraries, two open-source frameworks implementing several commonly used model-free deep RL algorithms. In particular, from SB3, we import the PPO algorithm^30^. For the mathematical modelling, we develop two models for the searcher shoot: (1) a model with the mechanics, but without the leaves (Me) and (2) a model with both the mechanics and the leaves (MeLe). We perform all the simulations with the discount factor $\gamma = 0.99$ (see Supplementary Material) and we train the models setting the number of episodes to 1 million.

**Figure 2:**
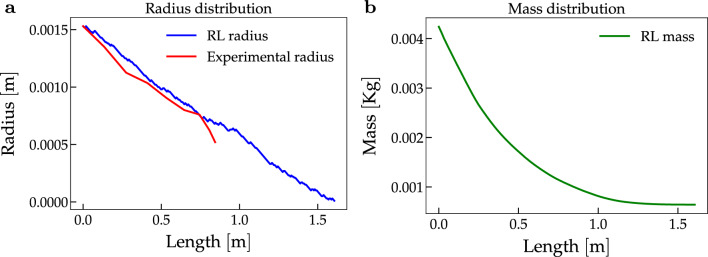
Radius and fresh mass distribution in the (Me) model.We show the radius and, consequently, the mass distribution obtained in the (Me) model after training. Specifically, in Fig. 2a, we compare the experimental radius (red) to the one obtained by our simulation (blue). Computing the relative error, we find that the discrepancy between the two radii is at most $8.55\%$ in segment [0, 0.868]. The value 0.868 represents the total length in meters of the shoot sample. In Fig. 2b, we show the simulated mass. The mass decreases smoothly and is approximately zero at the tip.

In Fig. 2, we plot the results of our simulations and we show the radius (Fig. 2a) and the mass distributions (Fig. 2b). We find that the radius decreases at each step, i.e. the agent chooses the actions leading to a smaller radius.

We compare our simulated scenario with samples S1–S5^15,27^. As we can notice in Fig. 2a, comparing the obtained radius distribution (in blue) with the experimental radius of sample S2 (in red), the relative error is $8.55\%$ in the segment [0, 0.868], which represents the length of the sample S2. In the Supplementary Material, we include the simulation results and the comparison with all the other four samples.

**Figure 3:**
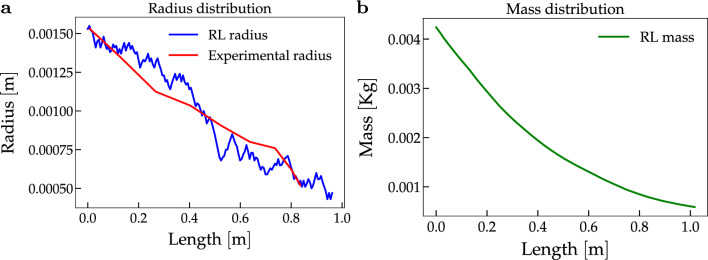
Radius and mass distribution in the (MeLe) model. We show the radius and, consequently, the mass distribution obtained in the (MeLe) model after the training. Specifically, in Fig. 3a, we compare the experimental radius (red) to the one obtained by our simulation (blue). Computing the relative error, we find that the discrepancy between the two radii is at most $10.28\%$ in the segment [0, 0.868]. The value 0.868 represents the total length in meters of the shoot sample. In Fig. 3b, we show the simulated mass. The mass decreases smoothly and is approximately zero at the tip.

Successively, we consider the (MeLe) model, where, in addition to the mechanics features, we model also the mass of the leaves along the shoot. In Fig. 3, we present the results of our simulations. Both the radius (Fig. 3a) and the mass distributions (Fig. 3b) are consistent with the results of the preceding model. Again, we compare the experimental radius (in red) of sample S2 with the one we derive using the (MeLe) model (in blue), and, by computing the relative error, we find that the discrepancy between the two radii is $10.28\%$ in segment [0, 0.868].

### Study on model sensitivity w.r.t leaves configuration

To evaluate the model’s adaptability to variations in leaf configuration, we conducted supplementary simulations introducing systematic changes in the length of the internode. In contrast to the original model, where the internode length is fixed at $0.13 \textrm{m}$, we explored a spectrum of lengths randomly generated within the range of [0.01, 0.5] (the length is generated at the beginning of a simulation and it doesn’t change within that simulation). The central aim of this investigation was to unveil the model’s sensitivity to leaf positioning, yielding nuanced insights. Noteworthy findings surfaced when the leaf cluster was positioned near the base of the shoot, revealing an average relative error of $7.43\%$ across 100 simulation rounds. In contrast, scenarios with internode lengths surpassing $0.2 \textrm{m}$ displayed a significantly higher average relative error, reaching $28\%$ over the same number of simulations.

**Figure 4:**
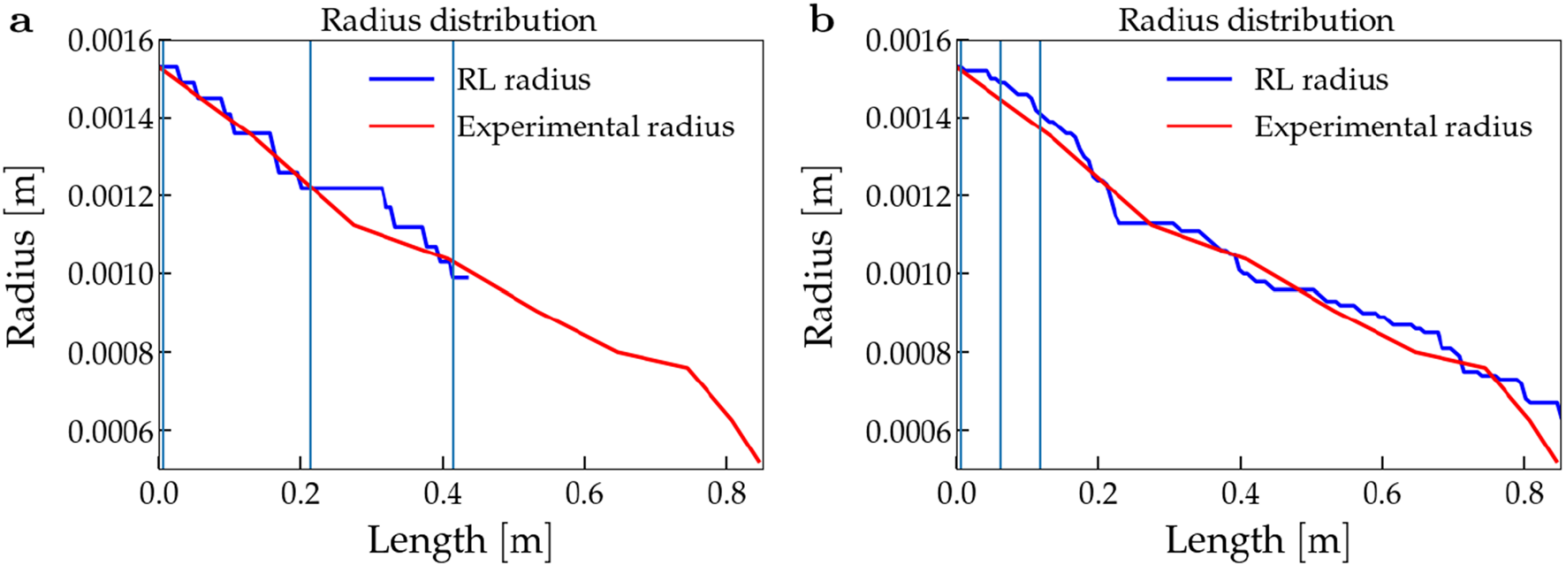
Example of different leaves configuration. We show the length and radius of the plant with different leaves’ configurations (vertical lines in light blue). As we can notice, in the best scenario the leaves are close to the base of the shoot.

In Fig. 4, we show an example of the worst (Fig. 4a) and the best leaves configuration (Fig. 4b). We show on the y-axis the radius distribution, on the x-axis the length of the plant’s shoot. The vertical lines in light blue represent the leaves’ position. In plot 4b, the internode is $0.0548 \textrm{m}$ long and the relative error in $7.29\%$. Instead, in plot 4a, the length of the internode is $0.2103 \textrm{m}$.

**Figure 5:**
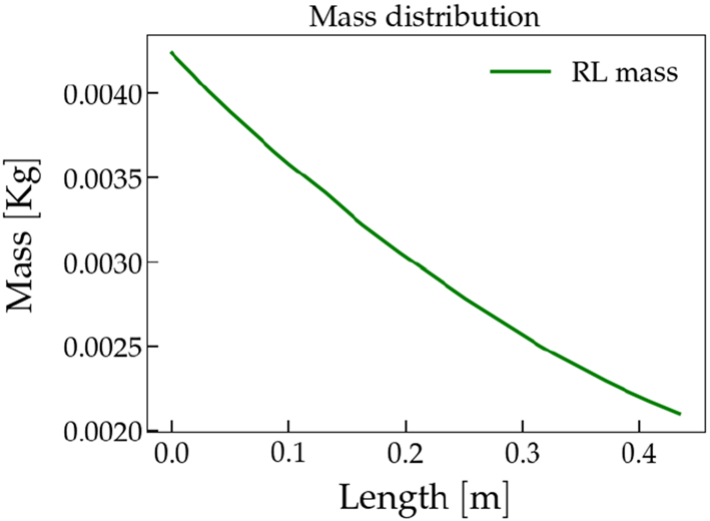
Mass distribution. We show the mass distribution associated to the example of the worst-case scenario of plot Fig. 4a. Here, on x-axis, we show the length of the shoot, on y-axis we show the mass of the plant. As we can notice, the mass does not go to zero, meaning that the plant’s growth is interrupted by the violation of the curvature threshold.

Furthermore, in the example of the worst-case scenario (4a), it becomes apparent that the mass of the leaves does not diminish to zero (see Fig.  5), indicating an interruption in the plant’s growth due to the violation of the curvature threshold. This behavior can be explained by considering the influence of the weight of the leaves on the slender shoots of the plant, which causes the violation.

### Model insights

**Figure 6:**
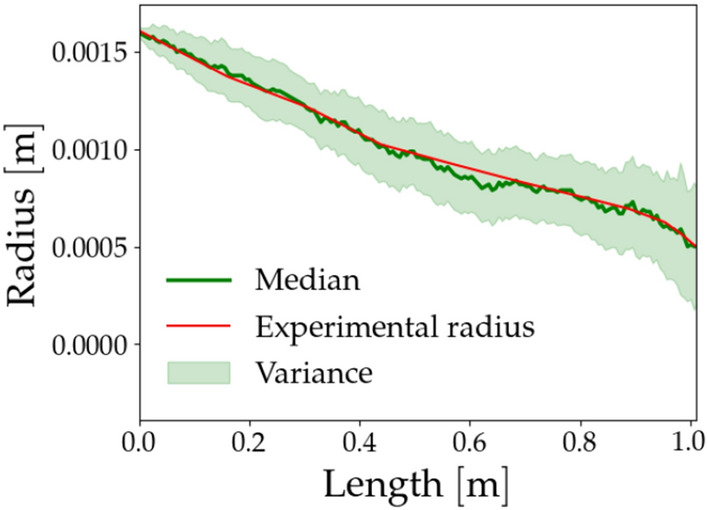
Median and variance computed over 100 simulations. We show the comparison with the samples S1.

To demonstrate the effectiveness of the model, Fig. 6 depicts the median and variance calculated from 100 simulations, alongside the experimental radii corresponding to sample S1. Additional sample plots can be found in the Supplementary Material for comparison.

## Discussion

The relative error between the measured radius (data provided by^15^) and the simulated samples is less than $15 \%$ for all the samples and in both cases with or without leaves (see Tables 1 and 2). Such a small relative error suggests that the optimal policy successfully reproduces the radial profile of the samples. Going into more detail, in the (Me) case with generic $c_2$ and $\psi _0$ (respectively, the constant for the curvature threshold and the ratio between curvature and the fourth power of the radius at the base of the shoot), the error is between 12.9 and $16.8 \%$ (see Table 1), with the exception of sample S1. This error decreases between 7.8 and $9.8 \%$ when the constants are sample-specific. In particular, we observe that the coefficient of variation of $c_2$ is about $35 \%$ (see Table 3), while the coefficient of variation of $\psi _0$ is much greater since it is approximately $74 \%$. This might imply that the environmental conditions have a relevant impact on the initial curvature of the shoot, while the stress threshold might be characteristic of the plant species. The difference between the length of the simulation and the experimental one, displayed in Fig. 2a, can be explained by the experimental error in the mass and density measurements, or by some assumptions on the model, such as a constant Young modulus all along the shoot. Regarding the simulations with the leaves, i.e. the (MeLe) model, the relative error gets worse if compared to the (Me) model (see Table 2). This drop in the accuracy of the model can be due to the fact that the agent has no control over the distribution of the leaves. Moreover, the constants $\psi _0$ and $c_2$ are estimated on a model based only on the optimisation of the mass of the main stem. Hence, effective implementation of the leaves would require a more accurate model; nevertheless, even in our approximation, we get an error between 7.4 and $14.1\%$. This result suggests that a functional advantage of the plant during the self-supporting stage is the optimization of the main stem’s mass. This is in line with the application of the behavioural ecology theoretical framework for plants (see^31^ for a survey on the behavioural ecology of climbing plants). In other words, according to this theory, plants have the capability to place their stems and other organs in accordance with optimal economic models. For instance, depending on the external supports in the surrounding environment, the risk of herbivory and the energetic stress, a shoot may delay leaf expansion or have short internodes. In our case, the plant has a limited amount of mass and can develop limited internal stress. A longer stem means, on one hand, a greater exploration capability, but on the other hand, a greater risk, since the structure is more fragile. Our RL environment gives a quantitative answer to the trade-off that the plant has to face, in the specific case of the *C. guianense* in its natural habitat. However, the generality of the equations at the base makes such an environment suitable for application to other plant species. Safety factors in terms of mechanical stability of stems and other plant organs is an informative way of relating size, length mass and stiffness to mechanical stability and height. The approach was not explored in this study primarily because of the longitudinal complexity and changing tissue patterning and stiffness (*E*) along the searcher stems. Our observations indicate that many searchers probably function under relatively low safety factors and rely on high stiffness values over developing wide diameters. Field observations even suggest that exceeding critical buckling length after crossing maximal reach gaps might be an advantage for contacting potential host supports just after a truly self-supporting phase^14^. In general, the use of a RL method to study a biological phenomenon can have some disadvantages, since it depends heavily on the concept of state and value function^32^. However, when properly formulated, it is a valuable instrument to predict and understand the behaviour of the phenomenon in consideration.

**Table 1:** The table reports the Relative Error with and without estimated parameters (EP), without considering the mass of the leaves. To show the strength of our results, we compute the Relative Errors by comparing our results with the experimental radii of the samples^15,27^. To understand the influence of two crucial parameters, $c_2$ and $\psi _0$, which are the curvature threshold and the initial curvature, respectively, we simulate our (Me) model by using their estimated values and an average value.

Sample	Relative error ($\%$) without EP	Relative error ($\%$) with EP
S1	8.6	13.6
S2	–	8.5
S3	16.8	8.2
S4	16.2	7.8
S5	12.9	9.8

**Table 2:** The table reports the Relative Error with estimated parameters, by considering the mass of the leaves. To show the strength of our results, we compute the Relative Errors by comparing our results ((MeLe) model) with the experimental radii of the samples^15,27^.

Sample	Relative error ($\%$)
S1	7.4
S2	10.2
S3	11.4
S4	14.1
S5	11.8

**Table 3:** The table reports the values of $\psi _0$, $c_2$, with average and coefficient of variation. The values of $\psi _0$ and $c_2$ are estimated utilizing the methods described in^33^.

Sample	$\psi _0$	$c_2$
S1	$- 5.9 e -12$	$8.7 e -4$
S2	$- 3.7 e -12$	$3.6 e -4$
S3	$- 7.8 e -12$	$4.8 e -4$
S4	$- 2.3 e -11$	$6.1 e -4$
S5	$- 1.1 e -11$	$4.9 e -4$
Average	$- 1 e-11$	$5.6 e -4$
Coeff. of variation	$73.61 \%$	$34.4 \%$

### Conclusion

In this work, we developed a RL environment called Searcher-Shoot to optimize the mass distribution along the main stem of a climbing plant during its self-supporting phase. At the base of the environment, there is a mechanical model that treats the plant’s stem as an elastic rod. We applied the Searcher-Shoot environment to the experimental data using two variants of the mechanical model: with or without leaves. The radii that we obtained from the numerical simulation are in accordance with the data, supporting the hypothesis that climbing plants apply the strategy of growing the stem as long as possible. Building an RL environment is a compelling strategy to understand complex systems biology behaviors, especially in the absence of crucial data required for the application of ML. The RL approach we employed in this work has yielded promising results, making us even more hopeful for future developments. In line with this work, we plan to build a more sophisticated model that can consider the curvature’s time development in addition to the optimal mass distribution. Indeed, plant movement is partially determined by the response to external signals. This response can be optimized to maximize (or minimize) a reward based on the signals themselves.

## Methods

### Notations for the mechanics of a planar elastic rod

Let $e_1,e_2,e_3$ a basis of orthonormal vectors for $\mathbb {R}^3$. We assume that the searcher shoot behaves like an inextensible and unshearable elastic rod^34^ confined in the plane spanned by the vectors $e_1$ and $e_2$. The centerline of this rod lies on the curve $\Gamma \subset \text {span}\{e_1,e_2\}$. We parametrize the curve $\Gamma$ with its arc length *s*, so that if the length of the curve is *L*, we have $s \in [0,L]$. We denote with $\underline{\Gamma }(s)$ the position in the plane of the point on $\Gamma$ whose arc length is *s*. With this parametrisation, $\partial _s\Gamma (s)$ represents the normal tangent vector to $\Gamma$ at the point $\underline{\Gamma }(s)$. We denote with $\theta (s)$ the angle between $\partial _s\Gamma (s)$ and the vector $e_2$. Consequently, $\theta '(s)$ is the curvature of $\Gamma$ at the point $\underline{\Gamma }(s)$.

We refer to $\Gamma$ as the *current* configuration, which corresponds to the actual shape of the rod when subject to external physical forces. To study the effects of gravity acting on that rod, we need to consider the *intrinsic* configuration, denoted as $\hat{\Gamma }$, which corresponds to the geometric curve assumed by the rod when there are no external forces acting on it. Since the rod is inextensible, the arc length parameter of $\hat{\Gamma }$ and $\Gamma$ is the same $s \in [0,L]$.

The difference between the curvature $\partial _s\hat{\theta }(s)$ of the intrinsic configuration and the curvature $\partial _s \theta (s)$ of the current configuration is proportional to the resultant moment (of force) *m*(*s*) acting at the point $\underline{\Gamma }(s)$ (and directed along $\underline{e}_3$) through the Euler-Bernoulli formula:

1 $$\begin{aligned} m(s) = - E(s)I(s)(\partial _s\theta (s) - \partial _s\hat{\theta }(s)). \end{aligned}$$

This relation holds because we are considering unshearable rods. With *E* we are denoting the Young’s modulus, which expresses the stiffness of the material, and with *I* the second moment of area of the cross-section (with respect to $e_3$). We assume that the rod is in elastic equilibrium, that is, all the internal forces and moments are in balance with the external forces and moments. In this framework, considering equation (1) and the gravity force as the only external force acting on the rod, we can write the following differential equation (see for instance^35^)

2 $$\begin{aligned} \partial _s(EI(\partial _s\hat{\theta } - \partial _s\theta ))(s) = \sin (\theta (s))g\int _s^L \rho _3(s') A(s') ds'. \end{aligned}$$

In this equation, *g* represents the gravity acceleration constant, $\rho _3(s)$ the volume density of the shoot/rod at the point $\underline{\Gamma }(s)$ and *A*(*s*) the cross-section area at that point.

We are now interested in computing the internal bending stresses. We know that the internal moment *m* is generated by the deflection from the intrinsic configuration $\hat{\Gamma }$. Indeed, this deflection generates an internal pressure called *stress*, that we denote with $\sigma$, and a deformation $\varepsilon$ of each element of the rod, called *strain*. Stresses and strains vary according to the position $\underline{\Gamma }(s)$ on the rod, and depend also on the position on the cross-section. Since the rod is ushearable, the cross-section is always orthogonal to the tangent vector $\partial _s \Gamma$. To describe the position of a generic point on the cross-section at $\underline{\Gamma }(s)$, we name

$$\begin{aligned} \beta (s) = \frac{\partial ^2_s\Gamma (s)}{|\partial ^2_s\Gamma (s)|} \in \text {span} \{ e_1, e_2\} \end{aligned}$$

the normal vector, which is orthogonal to $\partial _s\Gamma (s)$, and we consider the binormal vector

$$\begin{aligned} \tau (s) = \frac{\partial _s \Gamma \times \beta (s)}{|\partial _s \Gamma (s) \times \beta (s)|}, \end{aligned}$$

Then, the cross-section at the point $\underline{\Gamma }(s)$ is a subset *C*(*s*) of the plane $\text {span} \{ \beta (s), \tau (s) \}$ with the origin on the centerline. We define

$$\begin{aligned} C(s,z) = \{ w \in \mathbb {R} : \, \underline{\Gamma }(s) + z\beta (s) + w \tau \in C(s) \}. \end{aligned}$$

In this framework, the maximal bending stress at $\Gamma (s)$ results to be^36^

3 $$\begin{aligned} \begin{aligned} \sigma _m(s)&= \max \{|w| : \, C(s,w) \ne \emptyset \} \cdot \frac{m(s)}{I(s)} \\&= \max \{|w| : \, C(s,w) \ne \emptyset \} \cdot E(s) |\partial _s\theta (s) - \partial _s \hat{\theta }(s) |. \end{aligned} \end{aligned}$$

### Formulation of the models

We assume that the searcher shoot has a circular cross-section with radius *r* and that the Young’s modulus *E* is constant all along the shoot. So, we have

$$\begin{aligned} \begin{aligned} A(s)&= \pi r^2(s) \\ I(s)&= \frac{\pi }{4}r^4(s) \\ r(s)&= \max \{|w| : \, C(s,w) \ne \emptyset \} \end{aligned} \end{aligned}$$

and we name

$$\begin{aligned} u(s) = r^2(s), \, \psi (s) = u^2(s)(\partial _s \hat{\theta }(s) - \partial _s \theta (s)), \, \mu (s) = \int _s^L \pi \rho _3(s')u(s') ds' \end{aligned}$$

So, equation (2) can be rewritten as

4 $$\begin{aligned} {\left\{ \begin{array}{ll} \partial _s \psi (s) = c_1 \sin (\theta (s)) \mu (s) \\ \partial _s \theta (s) = \partial _s \hat{\theta }(s) - \frac{\psi (s)}{u^2(s)} \\ \partial _s \mu = - \pi \rho _3(s) u(s) \end{array}\right. } \end{aligned}$$

with

$$\begin{aligned} c_1 = \frac{4g}{\pi E}. \end{aligned}$$

These equations hold for a.e. $s \in [0,L]$. At the boundary of this domain, we assume (1) to know the angle at the base of the shoot $\theta (0) = \theta _0$; (2) that at the tip of the shoot, there is not any external weight so that the intrinsic curvature equals the current curvature. Using the functions defined above, this means $\psi (L) = 0$ and $\mu (L) = 0$; (3) the mass *M* of the whole shoot is known, so that $\mu (0) = M$.

The effectiveness of the self-sustaining behavior of the shoot can be quantitatively evaluated considering a threshold for the maximal internal stresses. In other words, we would like to find a distribution of the mass such that the maximal stress at each point of the shoot $\sigma _m(s)$ does not cross some fixed value $\bar{\sigma }$. Employing equation (Eq. 3), this means that any solution of system (Eq. 4) which satisfies the boundary conditions above discussed, has also to satisfy the condition

5 $$\begin{aligned} |\psi (s)| \le c_2 u^{3/2}(s) \text { for every } s \in [0,L] \end{aligned}$$

with

$$\begin{aligned} c_2 = \frac{\bar{\sigma }}{E}. \end{aligned}$$

We group all these considerations into the following problems.

**Problem of shoot growth with Mechanics** (Me). We want to find the maximal length *L* of the shoot for which there exists at each point a radius *r*(*s*) such that the following system is satisfied

Me $$\begin{aligned} {\left\{ \begin{array}{ll} \partial _s \psi (s) = c_1 \sin (\theta (s)) \mu (s) \\ \partial _s \theta (s) = \partial _s \hat{\theta }(s) - \frac{\psi (s)}{u^2(s)} \\ \partial _s \mu = - \pi \rho _3(s) u(s) \\ \psi (L) = 0 \\ \theta (0) = \theta _0 \\ \mu (0) = M, \, \mu (L) = 0 \\ |\psi (s)| \le c_2 u^{3/2}(s) \end{array}\right. }. \end{aligned}$$

**Problem of shoot growth with Mechanics and Leaves** (MeLe). In problem (Me) we consider just the weight of the main stem. However, in most of climbing plant species, a relevant part of the total biomass is due to the leaves. We assume that the leaves are not uniformly distributed along the shoot. On the contrary, we assume that they are located at intervals equally spaced. Moreover, we assume that the mass $m_{\text {lm}}$ of a single leaf at the point $\underline{\Gamma }(s)$ depends just on the shoot radius *r*(*s*) at $\underline{\Gamma }(s)$ (so $m_{\text {lm}} = m_{\text {lm}}(r(s))$). Let $d_{\text {lm}}$ the distance between two leaves locations and $n_{\text {lm}}$ the (fixed) number of leaves at each location. Then, we name

$$\begin{aligned} s_i = i \times d_{\text {lm}} \text { for } i = 1,..., \, N_{\text {lm}}, \end{aligned}$$

where $N_{\text {lm}}$ is the total number of leaves locations. Therefore, $s_i$ denotes the arc length of the *i*-th leaves location. Now, we want to compute how the weight of the leaves affects the shoot at the point $\underline{\Gamma }(s)$. To achieve this, we subtract from the total leaves mass $M_{\text {lm}}$ the mass of the leaves in the shoot portion between the base and $\underline{\Gamma }(s)$. We define

$$\begin{aligned} q_{\text {lm}}(s) = \left\lfloor \frac{s}{d_{\text {lm}}} \right\rfloor \end{aligned}$$

and

$$\begin{aligned} \text {RES}_{\text {lm}}(s) = M_{\text {lm}} - n_{\text {lm}} \sum _{i = 0}^{q_{\text {lm}}(s)}m_{\text {lm}}(r(s_i)), \end{aligned}$$

where the operation $\lfloor x \rfloor$ is the greatest integer lower or equal than *x*. Then, to take the leaves into account, we compute the gravity force acting at the point $\underline{\Gamma }(s)$ :

$$\begin{aligned} -g \left[ \int _s^L \rho _3(s') A(s') ds' + \text {RES}_{\text {lm}}(s) \right] e_2. \end{aligned}$$

This leads to another problem of length maximization. Like for the (Me) case, we want to find the maximal length *L* of the shoot for which there exists at each point a radius *r*(*s*) such that

MeLe $$\begin{aligned} {\left\{ \begin{array}{ll} \partial _s \psi (s) = c_1 \sin (\theta (s)) \left( \mu (s) + \text {RES}_{\text {lm}}(s) \right) \\ \partial _s \theta (s) = \partial _s \hat{\theta }(s) - \frac{\psi (s)}{u^2(s)} \\ \partial _s \mu = - \pi \rho _3(s) u(s) \\ \psi (L) = 0 \\ \theta (0) = \theta _0 \\ \mu (0) = M, \, \mu (L) = 0 \\ |\psi (s)| \le c_2 u^{3/2}(s) \end{array}\right. }. \end{aligned}$$

### Reinforcement Learning framework

We define the Searcher-Shoot environment as a Markov decision process problem (see Supplementary Material). Here, the agent is the liana’s searcher shoot, a structure that has presumably been selected during evolution to have the greatest reach to colonize its environment by adjusting on its mechanical properties and the taper of the shoot’s diameter. Specifically, the agent learns how to complete the task (i.e., the mass distribution) in the highest number of steps, choosing radius values does not generate internal stresses over a fixed threshold.

The fundamental elements of the framework are:

- **State and observations.** At each step, the agent, in the current state, can observe the mass, the radius, and the curvature before the next move. The choice of such a state is due to algorithmic and biological reasons. Indeed, in our algorithm, the agent controls the distribution of the mass by choosing the radius at each step. To this aim, the agent is able to observe the (remaining) mass and the radius. Regarding the curvature, it is a stop criterion and an index of efficiency. Moreover, plants are able to sense their own curvature through specialized fiber cells^37^. Plants are also able to sense their own inclination^38^, but we prefer to keep the number of observable variables as low as possible in order to reduce the computational effort of the algorithm.
- **Actions.** In this framework, the action space is discrete. In the (Me) and (MeLe) models, at each time step, the agent can select one action among eleven options: it can leave the radius value unchanged or it can increase (or decrease) the radius of a certain quantity ($\pm 1 \times 10^{-5}$, $\pm 2 \times 10^{-5}$, $\pm 3 \times 10^{-5}$, $\pm 4 \times 10^{-5}$, $\pm 5 \times 10^{-5}$). Of course, this selection will influence the mass distribution: intuitively, the larger the radius value, the larger the mass allocated in the next step.
- **Reward.** Every time the agent moves to the next step, it will receive positive feedback equal to $+ 1$. Whether the move ends with the total mass equivalent to or less than 0, or the condition on the curvature (Eq. 5) is violated, the reward is 0 and the algorithm stops.
- **Episode and Reset.** The episode does not have a fixed term. Instead, it ends whether the mass becomes zero or negative or the picked radius causes the curvature to violate condition (Eq. 5), as displayed in Algorithm 1 and 2. Then, we set the system parameters and the observation space to their initial values.

**Algorithm 1:**
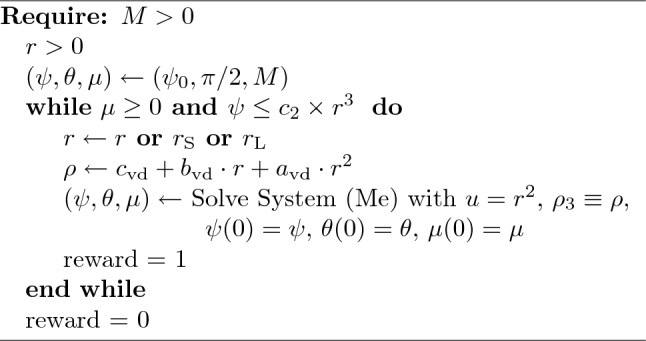
Algorithm of Shoot growth with mechanics.

**Algorithm 2:**
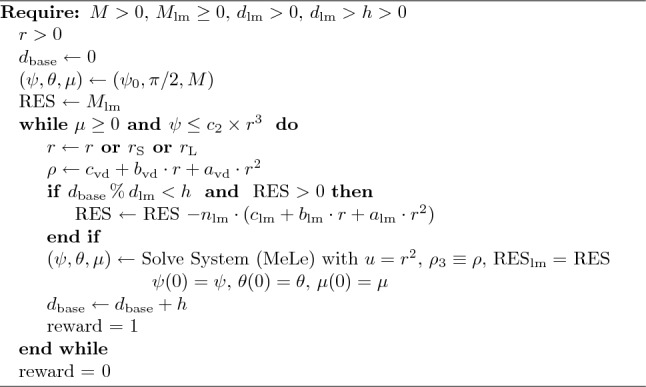
Algorithm of Shoot growth with mechanics and leaves.

### Models implementation and parameters

To begin with, we implement the System of equations (Me) in the Searcher-Shoot environment. In such a system, we consider *stress* and *strain* as factors responsible for its shaping, together with *gravity*, which acts as an external force on the plant’s structure, affecting its curvature. Moreover, the material *density* is a function of the radius *r*, defined as follows:

6 $$\begin{aligned} \rho _3 = c_{\text {vd}} + b_{\text {vd}} \cdot r + a_{\text {vd}} \cdot r^2. \end{aligned}$$

We use Algorithm 1 to clarify the approach we implemented. Starting from an initial configuration of curvature, angle and mass $(\psi _0, -\pi /2,M)$, and given an initial radius $R_0$, at each step of the algorithm the agent chooses how much to increase or decrease the current radius. Consequently, the total mass decreases and curvature and angle change accordingly to System (Me). This process is repeated until the mass vanishes or the curvature constraint is violated. The values of the constants $c_2$ and $\psi _0$ are estimated in two ways, leading to two groups of simulations. In the first group, $c_2$ and $\psi _0$ are the same for all the samples, while in the second group, they are estimated specifically for each sample by utilizing the method described in^33^.

Successively, we add the leaves’ mass contribution, which affects the plant’s weight remarkably. We implement the System of equations (MeLe), where we can notice that the effects of the leaves are visible on the curvature and, then, in the formulation of the equation of $\psi$. As for the material density, the mass of a single leaf depends on the radius *r* according to the following relation:

7 $$\begin{aligned} m_{\text {ml}} = c_{\text {lm}} + b_{\text {lm}} \cdot r + a_{\text {lm}} \cdot r^2. \end{aligned}$$

In Algorithm 2 we clarify how we implement our model in the RL context. The Algorithm iterations are similar to Algorithm 1, and in addition, we consider the mass of the leaves and check whether at the position of the agent there is a group of leaves or not. In Table 4, we include all the parameters used in the models.

**Table 4:** Parameters of the models. In the case of the parameters resulting from the fitting procedure, we report the source of the data on which functions (Eqs. 6, 7) are fitted.

Parameter	Description	Source
*g*	Gravity acceleration constant	^15,27^
*E*	Young’s modulus	^15,27^
*M*	Main stem freshmass	^15,27^
$c_2$	Curvature threshold	^33^
$\psi _0$	Initial curvature	^33^
$R_0$	Initial radius	^15,27^
$(a_{\text {vd}},b_{\text {vd}},c_{\text {vd}})$	Parameters for volume density fitting	^15,27^
$(a_{\text {lm}},b_{\text {lm}},c_{\text {lm}})$	Parameters for leaves mass fitting	^15,27^

### Statement on research methodology

The research methodology employed in this study adheres strictly to ethical and legal considerations associated with experimental research and field studies on plants. We affirm our compliance with relevant institutional, national, and international guidelines and legislation governing such research endeavors. Additionally, we emphasize our commitment to following the IUCN Policy Statement on Research Involving Species at Risk of Extinction and the Convention on the Trade in Endangered Species of Wild Fauna and Flora. This ensures the responsible and ethical conduct of our research with due consideration for the well-being of the studied plant species and the broader ecosystem.

### Supplementary Information

Supplementary Information.

## Data Availability

All data generated or analyzed during this study are included in this published article and in its Supplementary Material.
