# Supplementary material for: A Reinforcement Learning approach to study climbing plant behaviour
Source: Sci Rep. 2024 Aug 6;14:18222. doi: 10.1038/s41598-024-62147-3 (PMC11303795; doi:10.1038/s41598-024-62147-3)
Supplement: Supplementary file 1 — Supplementary Information. [file 41598_2024_62147_MOESM1_ESM.pdf]

Supplementary Material: A Reinforcement Learning approach to study climbing plant behaviour

Lucia Nasti[1], Giacomo Vecchiato[1], Patrick Heuret[2], Nicholas P. Rowe[2], Michele Palladino[1,3], and Pierangelo Marcati[1]

[1]Gran Sasso Science Institute, L'Aquila, Italy
[2]AMAP, Univ Montpellier, CIRAD, CNRS, INRAe, IRD, Montpellier, France
[3]DISIM, Department of Information Engineering, Computer Science and Mathematics, University of L'Aquila, Via Vetoio - 67100 L'Aquila, Italy

Abstract

A plant's structure is the result of constant adaptation and evolution to the surrounding environment. From this perspective, our goal is to investigate the mass and radius distribution of a particular plant organ, namely the *searcher shoot*, by providing a Reinforcement Learning (RL) environment, that we call Searcher-Shoot, which considers the mechanics due to the mass of the shoot and leaves. We uphold the hypothesis that plants maximize their length, avoiding a maximal stress threshold. To do this, we explore whether the mass distribution along the stem is efficient, formulating a Markov Decision Process. By exploiting this strategy, we are able to mimic and thus study the plant's behavior, finding that shoots decrease their diameters smoothly, resulting in an efficient distribution of the mass. The strong agreement between our results and the experimental data allows us to remark on the strength of our approach in the analysis of biological systems traits.

1 Supplementary Material

1.1 Reinforcement Learning

{SubSec: RL}

Supervised, Unsupervised, and Reinforcement learning are three paradigms of Machine Learning. While the first two approaches require examples and data (labeled or not) to extract information, RL models learn from interactions between an agent and an environment.

Specifically, the agent has an explicit goal, and to reach it, it can perform actions that influence the state of the environment, which has a set of immutable rules. The agent uses these interactions to adjust its behavior to complete its

task. Beyond these, four sub-elements characterize the RL system [1]: policy, reward, value function and model. The meaning of these sub-elements is the following:

- The *policy* represents how the agent chooses the action based on the current state;
- The *reward* is the goal of the RL problem. It is a feedback signal defining the good and bad events for the agent;
- The *value function* is the total amount of reward an agent can expect to accumulate over the future, starting from a specific state. It helps the agent to understand the long-term consequences of actions;
- The *model* of the environment is an optional representation of the environment, which allows the planning of possible future situations before their experience.

We can express an RL problem using the mathematical formalism of the Markov Decision Process (MDP), used to study the control of sequential decisions that can influence states and future rewards. An MPD is a tuple $\mathcal{M} = \langle \mathcal{S}, \mathcal{A}, \mathcal{R}, \mathcal{P}, \gamma \rangle$ where $\mathcal{S}$ and $\mathcal{A}$ are the state and the action space, respectively; $\mathcal{R}$ is the reward function $\mathcal{R} : \mathcal{S} \times \mathcal{A} \mapsto \mathbb{R}$, representing the immediate reward; and, $\mathcal{P}$ is the transition function $\mathcal{P} : \mathcal{S} \times \mathcal{S} \times \mathcal{A} \mapsto [0, 1]$, and so the probability to move from a state to another having chosen an action. Finally, γ represents the discount factor, namely the chance for the agent to choose between an instant (short-sighted agent) and a future reward (farsighted agent).

Briefly, at each time step t, in a state $s_t \in \mathcal{S}$, an agent interacts with the environment and chooses an action $a_t \in \mathcal{A}$, which leads to a reward $r_{t+1} = \mathcal{R}(s_t, a_t)$ and a transition to a new state $s_{t+1} \in \mathcal{S}$. The probability to reach the state s_{t+1} is given by $\mathcal{P}(s_t, s_{t+1}, a_t)$.The choice of the action a_t relies on the policy adopted by the agent. Formally, a policy is a function $\pi : \mathcal{S} \times \mathcal{A} \mapsto [0, 1]$ which gives the probability of choosing an action $a \in \mathcal{A}$ knowing that the agent is in the state $s \in \mathcal{S}$. The goal is to maximize the total reward, learning a policy.

1.1.1 Proximal Policy Optimization

{SubSec: PPO Algorithm}

We can divide the RL algorithms into two main categories: *Model-Based*, in which a system uses a predictive model of the world to choose the best action (i.e., the algorithm exploits the knowledge of a Markov Decision Process); *Model-free*, in which the agent learns a value function or a policy by interacting with the environment [2].

Having a model means relying on a function that predicts the future states and rewards, allowing the agent to plan by thinking ahead and explicitly deciding between its options. Instead, the absence of it means that the agent uses only the current state and its experience to learn.

In our work, we exploit the model-free approach in the form of the Proximal Policy Optimization (PPO) algorithm, introduced by Schulman et al. [3] in 2017. PPO is a policy gradient, on-policy algorithm, meaning that the algorithm is searching for an approximation of the best policy through a parameter θ, and each step for the upgrade of the policy π_θ relies on a sampling based on π_θ itself. There are two primary variants: PPO-Penalty and PPO-Clip, which we use.

In the PPO-Clip approach, the update of the parameter θ_k to θ_{k+1} relies on the maximisation of the following surrogate objective:

$$\theta_{k+1} = \arg\max_{\theta} \mathop{\mathbb{E}}_{(s,a)\sim\pi_{\theta_k}} \left[L(s, a, \theta_k, \theta) \right]$$

with

$$L(s, a, \theta_k, \theta) = \min \left(\frac{\pi_\theta(a|s)}{\pi_{\theta_k}(a|s)} A^{\pi_{\theta_k}}(s, a), g(\epsilon, A^{\pi_{\theta_k}}(s, a)) \right),$$

and

$$g(\epsilon, A) = \begin{cases} (1+\epsilon)A & A \geq 0 \\ (1-\epsilon)A & A < 0. \end{cases}$$

$A^{\pi_{\theta_k}}$ is the advantage function related to the policy π_{θ_k},

$$\mathop{\mathbb{E}}_{(s,a)\sim\pi_{\theta_k}}$$

stands for the average with respect to (s, t). With this notation, the actions a are distributed according to the policy π_{θ_k} and the states s follow the stationary distribution of the Markov chain for the policy π_{θ_k}. The proof of the convergence of this method is in [4]. This approach tries to increase the probability of taking the best action without moving too far from the current policy, avoiding the system collapse (trust region approach [5]). Indeed, in L the hyperparameter ϵ represents how far the new policy can be from the old one. If A is positive, the picked action is better than the expectations, and it becomes more likely to choose it again. Otherwise, if A is negative, the picked action will be less called. The minimum and the function g limit the policy change by imposing the probability ratio to stay within an interval of amplitude 2ϵ of around 1.

1.2 Derivation of the model

In this section, we describe in detail the derivation of the model. Some excellent guidelines for elastic rods and material mechanics can be found in [6, 7].

Consider a planar elastic rod Γ, $\{\underline{d}_1, \underline{d}_2, \underline{d}_3\}$ subject to an external force F and an external moment L. The balance between the internal force n and the internal moment m with F and L is expressed by the following equations:

$$\begin{cases} \partial_s \underline{n} + \underline{f} = 0 \\ \partial_s \underline{m} + \partial_s \underline{\Gamma} \times \underline{n} + \underline{l} = 0 \end{cases} \quad (1)$$ {eq:balance}

where f and l represents respectively the external force F and the external moment L per unit of length. In other words, we write $f = \partial_s F$ and $l = \partial_s L$.

Since we are just considering the gravity force, we employ the plane coordinates $\{\underline{e}_1, \underline{e}_2\}$ to recast the first equation of system (1):

$$\partial_s \begin{bmatrix} n_1 \\ n_2 \end{bmatrix} + \begin{bmatrix} 0 \\ -g\rho_3(s)A(s) \end{bmatrix} = 0.$$

Here g is the gravity acceleration constant, $\rho_3(s)$ is the volume density of the elastic rod and $A(s)$ is the area of the cross-section of the rod at the point $\underline{\Gamma}(s)$. We assume that there are no internal forces acting at the tip of the rod. So, we get

$$n_1 \equiv 0\,,\; n_2(s) = -g\int_s^L \rho_3(s')A(s')ds'. \tag{2}$$ {eq:balance_force}

The internal moment m per unit of length must be balanced with the moment per unit of length generated by the internal force n (second equation of system (1) with $l = 0$). This gives the relation:

$$\partial_s m(s) + \sin(\theta(s))n_2(s) - \cos(\theta(s))n_1(s) = 0. \tag{3}$$ {eq:balance_moment}

The combination of the Euler-Bernoulli equation for an elastic rod (Equation (1) in the main text), (2) and (3) give the balance equation (2) in the main text.

Now, we want to prove the equation for the maximal stress (Equation (3) of the main text). We assume that stress σ and strain ε are proportional:

$$\sigma(s, z) = E(s)\varepsilon(s, z).$$

We recall that z represents the distance from the centreline along $\beta(s)$ on the cross-section $C(s)$. We also assume that the strain ε has the following form:

$$\varepsilon(s, z) = \alpha(s)z,$$

where $\alpha(s)$ is a proportionality constant that may vary along the rod. Since the stress $\sigma(s, z)$ is applied to the infinitesimal strip $\mathcal{L}(C(s, z))dz$, where $\mathcal{L}$ is the length (to be more precise, the Lebesgue measure) of $C(s, z)$, the internal moment acting on the cross-section $C(s)$ with respect to its center is

$$\begin{aligned} m(s) &= \int_{\mathbb{R}} z\sigma(s, z)\mathcal{L}(C(s, z))dz \\ &= E(s)\alpha(s)\int_{C(s)} z^2 dzdw \\ &= E(s)\alpha(s)I(s). \end{aligned}$$

This gives the equation for the maximal stress because we get

$$\frac{m(s)}{I(s)} z = \sigma(s, z).$$

Consequently, the maximal stress is at the edge of the cross-section, with $z = \max\{|y| \,:\, C(s, y) \neq \emptyset\}$.

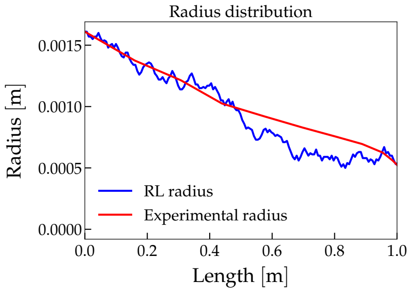

{supplementary figure 1}

Supplementary Figure S1: Radius distribution comparison between (Me) model (with estimated parameters) and experimental radii. The plot shows the sample S1.

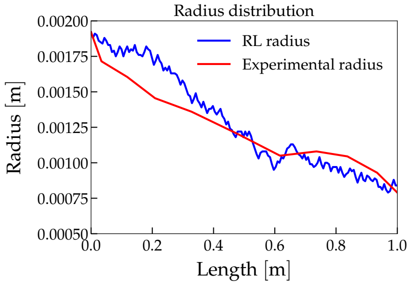

{supplementary figure 2}

Supplementary Figure S2: Radius distribution comparison between (Me) model (with estimated parameters) and experimental radii. The plot shows the sample S3.

1.3 Further Simulations and Tables

In Supplementary Figures S1-S2-S3-S4 we show the performance of our (Me) model w.r.t. the experimental radii of the samples, which appear in [8, 9].

In Supplementary Figures S5-S6-S7-S8, we show the performance of our ($MeLe$) model w.r.t. the experimental radii of the samples, which appear in [8, 9].

1.4 Simulation Design and Implementation

This section takes a closer look at the choices we made when designing our simulation. Two key decisions stand out: the deliberate selection of unique random seeds for each simulation, ensuring robustness, and the visualization of learning curves. These choices aren't just technical details; they're the foundation of a robust simulation environment.

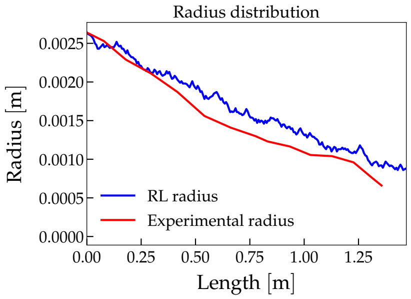

{supplementary figure 3}

Supplementary Figure S3: Radius distribution comparison between (Me) model (with estimated parameters) and experimental radii. The plot shows the sample S4.

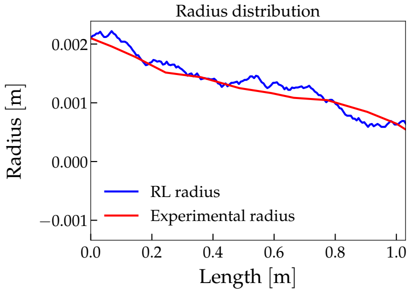

{supplementary figure 4}

Supplementary Figure S4: Radius distribution comparison between (Me) model (with estimated parameters) and experimental radii. The plot shows the sample S5.

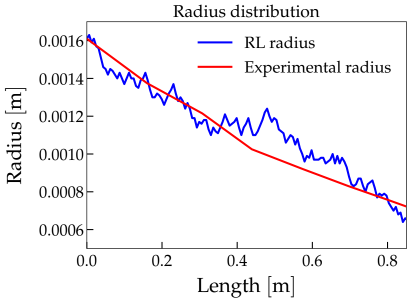

{supplementary figure 5}

Supplementary Figure S5: Radius distribution comparison between ($MeLe$) model (with estimated parameters) and experimental radii. The plot shows the sample S1.

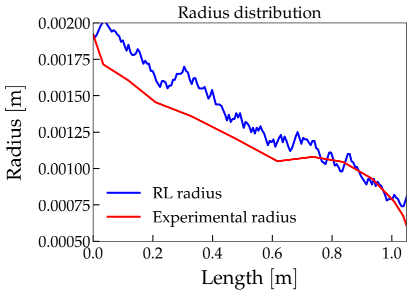

{supplementary figure 6}

Supplementary Figure S6: Radius distribution comparison between ($MeLe$) model (with estimated parameters) and experimental radii. The plot shows the sample S3.

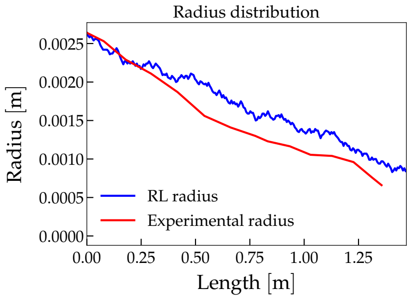

{supplementary figure 7}

Supplementary Figure S7: Radius distribution comparison between ($MeLe$) model (with estimated parameters) and experimental radii. The plot shows the sample S4.

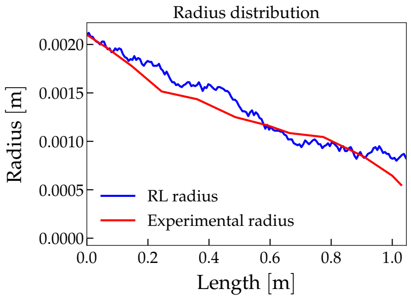

{supplementary figure 8}

Supplementary Figure S8: Radius distribution comparison between ($MeLe$) model (with estimated parameters) and experimental radii. The plot shows the sample S5.

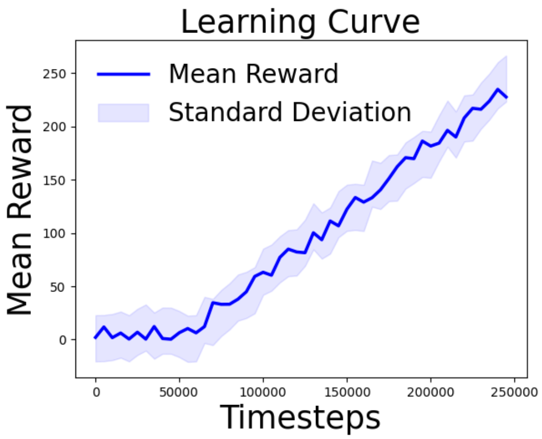

{supplementary figure 9}

Supplementary Figure S9: Learning curve. We show the learning curve of our RL model with leaves. On the x-axis, we plot the timesteps (meaning the interaction between the agent and the environment), and on the y-axis the mean reward. We compute the learning curve over 100 simulations.

Random seed

To observe how the learning process adapts, we enable the random number generator to initialize differently in each run. By avoiding the specification of a random seed, we can evaluate the algorithm's overall behavior across diverse initial conditions. Indeed, in this way, we can assess the algorithm's general behavior across different initial conditions. However, during the initial stages of our experiments, we used a fixed random seed (set to 42) to assist us in understanding the learning process and to aid us in debugging. This was a conscious decision that we made to closely analyze the behavior of the algorithm under controlled conditions, ensuring that the results could be replicated and allowing for effective troubleshooting.

Plot of learning curve

A learning curve is a graphical representation that illustrates how an agent's performance evolves or training steps. The curve typically depicts a performance metric, such as the cumulative rewards or the average rewards obtained by the agent as it interacts with the environment and learns from its experiences.

In Figure S9, while the y-axis depicts the mean reward obtained by the agent during its interactions with the environment, the x-axis represents the progression of training steps (timesteps). Here, timesteps refer to a discrete unit of time or a single step in the interaction between the agent and its environment.

The learning curve illustrates a positive trajectory where the mean reward consistently rises with increasing timesteps. This upward trend signifies that the PPO algorithm is effectively learning and improving its performance in the given

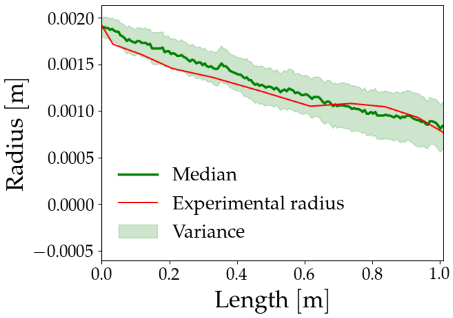

{supplementary figure 10}

Supplementary Figure S10: Median and variance computed over 100 simulations. The plot shows the comparison with the sample S2.

environment. The random noise reflects the stochastic nature of the learning process.

In generating the learning curve presented in our supplementary material, we deliberately chose to focus on the environment with mechanics and leaves (MeLe), which represents the more complex variant within our experimental set. The rationale behind this decision stems from the recognition that the learning curve's dynamics may be influenced by the complexity of the model. By showcasing the algorithm's performance in a more intricate environment, we aim to provide a robust assessment of its adaptability and learning capabilities. If the learning curve indicates positive progress in this complex setting, it offers a compelling indication that the algorithm can effectively navigate and learn in challenging conditions. Consequently, this obviates the necessity for simulating the simpler environment, streamlining our evaluation process.

Model insights

To illustrate the efficacy of the model, in Supplementary Figures S10-S11-S12-S13, we show the median and variance, computed across 100 simulations, juxtaposed with the experimental radii corresponding to the four distinct samples (S2, S3, S4, S5).

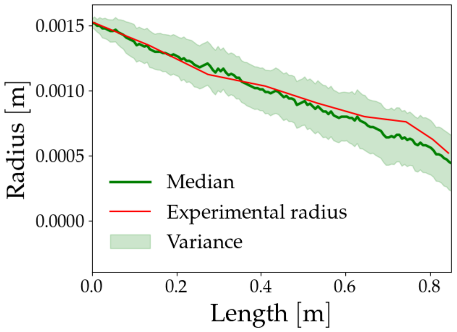

{supplementary figure 11}

Supplementary Figure S11: Median and variance computed over 100 simulations. The plot shows the comparison with the sample S3.

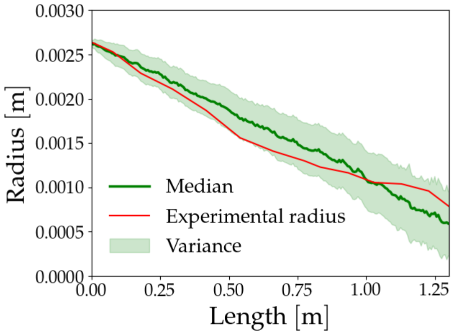

{supplementary figure 12}

Supplementary Figure S12: Median and variance computed over 100 simulations. The plot shows the comparison with the sample S4.

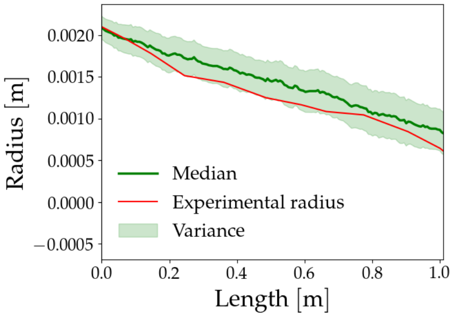

{supplementary figure 13}

Supplementary Figure S13: Median and variance computed over 100 simulations. The plot shows the comparison with the sample S3.

References

[1] Richard S Sutton and Andrew G Barto. *Reinforcement learning: An introduction*. MIT press, 2018.

[2] Tingwu Wang et al. “Benchmarking model-based reinforcement learning”. In: *arXiv preprint arXiv:1907.02057* (2019).

[3] John Schulman et al. “Proximal policy optimization algorithms”. In: *arXiv preprint arXiv:1707.06347* (2017).

[4] Markus Holzleitner et al. “Convergence proof for actor-critic methods applied to ppo and rudder”. In: *Transactions on Large-Scale Data-and Knowledge-Centered Systems XLVIII: Special Issue In Memory of Univ. Prof. Dr. Roland Wagner*. Springer, 2021, pp. 105–130.

[5] Andrew R Conn, Nicholas IM Gould, and Philippe L Toint. *Trust region methods*. SIAM, 2000.

[6] Alain Goriely. *The mathematics and mechanics of biological growth*. Vol. 45. Springer, 2017.

[7] Barry J Goodno and James M Gere. *Mechanics of materials*. Cengage learning, 2020.

[8] Tom Hattermann et al. “Mind the gap: reach and mechanical diversity of searcher shoots in climbing plants”. In: *Frontiers in Forests and Global Change* 5 (2022).

[9] Giacomo Vecchiato et al. “A 2D model to study how secondary growth affects the self-supporting behaviour of climbing plants”. In: *PLOS Computational Biology* 19.10 (2023), e1011538.
